# Safety and Immunogenicity of a Canine Distemper DNA Vaccine Formulated with Lipid Nanoparticles in Dogs, Foxes, and Raccoon Dogs

**DOI:** 10.3390/vaccines13060614

**Published:** 2025-06-06

**Authors:** Hong Huo, Han Wang, Shulin Liang, Zilong Wang, Jinming Wang, Qingzhu Wang, Chan Li, Yuting Tao, Jinying Ge, Zhiyuan Wen, Jinliang Wang, Weiye Chen, Xijun Wang, Lei Shuai, Zhigao Bu

**Affiliations:** 1 State Key Laboratory for Animal Disease Control and Prevention, Harbin Veterinary Research Institute, Chinese Academy of Agricultural Sciences, Harbin 150069, China; huohong617@163.com (H.H.); wh_0614@163.com (H.W.); wangjinming1005@163.com (J.W.); ooooszh1@163.com (Y.T.); gejinying@caas.cn (J.G.); wenzhiyuan@caas.cn (Z.W.); wangjinliang@caas.cn (J.W.); chenweiye@caas.cn (W.C.); wangxijun@caas.cn (X.W.); 2 Harbin Enwei Biopharmaceutical Co., Ltd., Harbin 150069, China; liangshulin86@163.com (S.L.); zhuzi-1984@163.com (Q.W.); lichan@cspc.cn (C.L.); 3 CSPC Zhongqi Pharmaceutical Technology Shijiazhuang Co., Ltd., Shijiazhuang 050031, China; wzlchandler@163.com; 4 Jiangsu Co-Innovation Center for Prevention and Control of Important Animal Infectious Diseases and Zoonoses, Yangzhou University, Yangzhou 225009, China

**Keywords:** canine distemper, F protein, H protein, lipid nanoparticles, DNA vaccine

## Abstract

Background: canine distemper (CD) is a highly contagious and fatal disease caused by canine distemper virus (CDV), posing a significant threat to carnivores. New CDV strain circulation and multi-species infection may lead to the potential dilemma of safety concern and insufficient efficacy of the commercial modified live vaccines. Safe and effective vaccines for canine and wildlife prevention of CD need to be continuously updated and developed. Methods: we developed two DNA vaccines, p17F-LNP and p17H-LNP, encoding the fusion protein (F) or hemagglutinin protein (H) of a field CDV strain (HLJ17) and encapsulated in lipid nanoparticles (LNPs). Serum neutralizing antibody (NAb) was evaluated via neutralization tests, and mouse serum cytokine detection were evaluated via ELISA. Results: immunization of p17F-LNP and p17H-LNP monovalent or bivalent were safe, and induced robust CDV NAb and cytokine responses in mice. LNP encapsulation improved immune responses compared to naked DNA formulation, and the bivalent formulation of p17F-LNP and p17H-LNP (p17F/H-LNP) exhibited synergistic effects with a high level of immune responses. Moreover, two doses of p17F/H-LNP induced long-lasting CDV NAb for over 300 days in dogs, and prime and boost NAb responses in foxes and raccoon dogs. Conclusions: the preliminary findings provided here warrant further development of the p17F/H-LNP vaccine for animal targets against CDV infection.

## 1. Introduction

Canine distemper (CD) is an acute, highly contagious, and fatal infectious disease caused by the Canine Distemper Virus (CDV) [1]. Clinically, CD is characterized by biphasic fever, acute catarrhal rhinitis, pneumonia, conjunctivitis, and neurological damage [2]. CDV is globally distributed and affects a wide range of hosts, including various terrestrial carnivores such as members of the *Canidae*, *Procyonidae*, *Ursidae*, *Felidae*, *Viverridae*, *Hyaenidae*, and *Mustelidae* families [3]. In addition to common susceptible hosts such as dogs, ferrets, foxes, and raccoons, CDV poses a severe threat to endangered species such as the giant panda and tiger [4,5]. Vaccination is the primary method for CD prevention and control. However, currently commercial vaccines against CD seem ineffective in the presence of maternal antibodies and may induce fatal disease in some wildlife species [5,6], or may induce immunosuppression or neurological complications [7]. Outbreaks of CD continue in some countries because of the unsatisfactory effectiveness of the vaccines due to the low levels of immunity and short duration [8,9,10]. DNA vaccines, which induce both humoral and cellular immune responses through recombinant DNA, offer advantages such as safety, efficiency of long duration, low production costs, and simple manufacturing processes [11]. The use of lipid nanoparticles (LNPs) as a delivery system could enhance the transfection efficiency and immunogenicity of DNA vaccines [12], making them a promising alternative for CD prevention.

CDV is a member of the *Paramyxoviridae* family and the *Morbillivirus* genus. Its genome encodes six structural proteins, including nucleoprotein (N), phosphoprotein (P), matrix protein (M), fusion protein (F), hemagglutinin protein (H), and large polymerase protein (L) [13,14]. The H protein plays a crucial role in the viral attachment of host cell, while the F protein facilitates membrane fusion and plays a role in triggering both cellular and humoral immune responses against CDV, making them key targets for neutralization [15,16]. The F protein is initially synthesized as an inactive precursor, F_0_, which is then cleaved into active F_1_ and F_2_ subunits by intracellular proteases. These subunits mature in the Golgi apparatus and are transported to the cell surface, where they form a disulfide bond [17,18]. The F protein is highly conserved across different CDV strains, and even within the morbillivirus family, whereas the H protein exhibits considerable genetic variability, which can affect virulence and antigenicity [19]. Due to its genetic diversity, CDV is classified into numerous distinct lineages based on the variations in the H gene sequence [20]. The H protein binds to cell receptors, triggering a conformational change in the F protein, which promotes the fusion of the viral and cell membranes [21]. The two glycoproteins of CDV, H and F, are the primary antigenic proteins involved in the infection process and vaccine development.

In this study, we developed CDV DNA vaccines based on the F or H protein gene of a field CDV strains (HLJ17) derived from a dog with clinical symptoms of CD. The DNA vaccines were encapsulated in lipid nanoparticles (LNPs) as DNA-LNP formulation to enhance the immunogenicity and delivery efficiency. We preliminarily demonstrated the safety and immunogenicity in mice and immune persistence in dogs, and the immunogenicity in wildlife species such as foxes and raccoon dogs. Our preliminary results imply that the CDV DNA-LNP strategy can be developed as a prophylaxis against CDV infections.

## 2. Materials and Methods

### 2.1. Cells and Virus

Vero cells (ATCC, CCL-81) and HEK-293 cells (ATCC, CRL-1573) were cultured in Dulbecco’s modified Eagle’s medium (DMEM, Gibco, Waltham, MA, USA) supplemented with 10% fetal bovine serum (FBS, ExCell, Suzhou, China). Recombinant CDV expressing enhanced green fluorescent protein (rCDV-eGFP) based on the CDV/R-20/8 vaccine strain was propagated and titrated in Vero cells [22]. The virus was stored at −70 °C until use.

### 2.2. Construction of Recombinant Plasmids

A field CDV strain (HLJ17) from a dog with clinical symptoms of CD was isolated in 2017. The F and H protein genes (HLJ17H and HLJ17F) of HLJ17 were sequenced, and then we performed the sequence alignment and phylogenetic tree analysis based on sequences of the representative CDV strains in the GenBank by BLASTN program and MEGA11.0.13 software. The HLJ17H and HLJ17F were optimized for mammalian codon usage and synthesized to identify as target genes for vaccine development, and then cloned with restriction enzyme recognition sites Kpn I/Nhe I into the pCAGGS plasmid vector with the SV40 site deleting (pCAGGSΔSV40) and *Kanamycin* resistance, designated as recombinant plasmids p17F or p17H, respectively.

### 2.3. Plasmid DNA Identification

Large-scale preparation of the recombinant plasmids was performed using the PureLink™ HiPure Plasmid Maxiprep Kit (Invitrogen, Waltham, MA, USA) for subsequent identification, including double digestion, indirect immunofluorescence assay (IFA) and Western blot. The recombinant plasmids were digested with Kpn I (NEB, Beijing, China) and Nhe I (NEB, Beijing, China) restriction enzymes, followed by agarose gel electrophoresis to confirm successful cloning. A total of 1 μg of p17F or p17H was transiently expressed per well in HEK-293 cells in a 6-well plate using the ExFect Transfection Reagent (Vazyme, Nanjing, China) following the instructions. After 48 h of transfection, the cells were then performed IFA with dog anti-CDV serum as primary antibody and an FITC-conjugated rabbit anti-dog antibody as second antibody, or performed Western blot with dog anti-CDV serum as primary antibody and an IR-labeled rabbit anti-dog IgG as second antibody. The expression of CDV F and H proteins was analyzed using the EVOS FL cell imaging system (Thermo Fisher Scientific, Waltham, MA, USA) in IFA assay, and the Odyssey CLx Imaging System and Image Studio 5.2.5 software (LI-COR Biosciences, Lincoln, NE, USA) in western blot.

### 2.4. DNA-LNP Vaccine Preparation

The DNA-LNP vaccines were formulated as lipid nanoparticles (LNPs) using microfluidic technology as described previously [23]. Recombinant plasmids p17F and p17H were combined with a lipid mixture containing ethanol, phospholipids, ionizable lipids, DSPC, and cholesterol, designated as p17F-LNP and p17H-LNP. The lipids were dissolved in ethanol at a molar ratio of 50:10:38.5:1.5 to create the lipid component. The nucleic acids were dissolved in a pH 4.0 buffer comprising citric acid and sodium chloride. The organic and aqueous solutions were combined utilizing a T-junction mixer at a flow rate of 1:3 (amine to phosphate ratio (N/P) of 6). The isovolumetric TFF method was employed to maintain a constant flow rate of the permeated liquid and the addition of the buffer, keeping the retentate volume remained constant and the inlet pressure at 0.5 bar. The total mixing flow rate was 12 mL/min. The DNA-LNP candidate vaccines, p17F-LNP and p17H-LNP, were formulated and stored at −20 °C until use.

### 2.5. DNA-LNP Vaccine Determination

The DNA-LNP formulations were characterized for DNA concentration, particle size, size distribution, encapsulation efficiency, and in vitro [23]. The DNA-LNPs were solubilized in a chloroform and methanol mixture, and the DNA concentration was determined by quantifying the ultraviolet (UV) absorbance at 260 nm. The size of the DNA-LNPs was assessed by dynamic light scatter (DLS) on a Zetasizer Nano-ZS (Malvern, Worcestershire, UK). The DNA encapsulation efficiency was determined using the Quant-iT^TM^ dsDNA HS Reagent (Life Technologies, Carlsbad, CA, USA) following the instructions. The procedure entailed incubating the DNA-LNPs at room temperature for 2 min. The fluorescence was measured using a microplate reader at a standard fluorescein wave lengths (Excitation/Emission: 480/530 nm). The in vitro activity was performed using IFA analysis with 0.5 μg p17F-LNP, p17H-LNP, or bivalent formulations incubation per well in HEK-293 cells in a 24-well plate, and measured at the ratio of transfected fluorescence-positive cells using the EVOS FL cell imaging system (Thermo Fisher Scientific, USA) and IMAGE J 1.54d software.

### 2.6. Ethics Statement

All animal experiments were performed strictly following the Guide for the Care and Use of Laboratory Animals of the Ministry of Science and Technology of China, and approved by the Committee on the Ethics of Animal Experiments of the Harbin Veterinary Research Institute (HVRI), Chinese Academy of Agricultural Sciences (CAAS). Experiments were performed by trained personnel under biosafety level (BSL)-2 and animal biosafety level (ABSL-2) conditions.

### 2.7. Mouse Studies

For safety and immunogenicity evaluation, 6-week-old female mice (Vital River, Beijing, China) were randomly assigned to into 7 groups, and 6 mice were used for each group. Six groups of mice were incubated intramuscularly (*i.m.*) with 30 μg p17F, p17H, p17F-LNP, p17H-LNP, or bivalent formulation of 30 μg p17F and p17H (p17F/H), or 30 μg p17F-LNP and p17H-LNP (p17F/H-LNP) in a 0.1-mL volume twice over 21-day intervals. A group was injected with 0.1 mL PBS as a parallel control. Mice were observed daily for body weight changes for 21 days after prime immunization. Sera were collected to detect the CDV neutralizing antibody (NAb) response based on the rCDV-eGFP strain on days 21, 35 and 60 after prime immunization.

For immunogenicity evaluation of the applied dose of DNA-LNP formulation, 4 groups of 6-week-old female mice (Vital River, Beijing, China) were *i.m.* incubated with 10 μg p17F-LNP, p17H-LNP, bivalent formulation of 10 μg p17F-LNP and p17H-LNP (p17F/H-LNP) or PBS in a 0.1-mL volume twice over 21-day intervals. Mice were observed daily for body weight changes for 21 days after prime immunization. Sera were collected to detect the CDV NAb and cytokine responses at different time points after prime immunization.

### 2.8. Dog Studies

Ten 3-month-old beagle dogs (Xiling Cape, Jinan, China) were confirmed to be serologically negative for CDV, and randomly assigned to into 2 groups. Five dogs were *i.m.* incubated with bivalent formulation of 100 μg p17F-LNP and p17H-LNP (p17F/H-LNP) in a 0.1-mL volume twice over 21-day intervals. The other five dogs were *i.m.* received an approved CDV modified live vaccine MLV (Zoetis, Lincoln, NE, USA) twice at the recommended dose over 21-day intervals, and boost at day 120 after prime immunization. The dogs were observed daily for clinical status, and bled for a period of 300 days after prime immunization for CDV NAb assessment.

### 2.9. Fox and Raccoon Dog Studies

Four 4-month-old farmed blue foxes and four 4-month-old farmed raccoon dogs from local farms were confirmed to be serologically negative for CDV. Foxes and raccoon dogs were *i.m.* incubated with bivalent formulation of 100 μg p17F-LNP and p17H-LNP (p17F/H-LNP) in a 0.1-mL volume twice over 21-day intervals. The animals were observed daily for clinical status, and sera were collected on day 14, 21 and 35 after prime immunization for CDV NAb assessment.

### 2.10. Serum CDV NAb Detection

Animal serum samples were thawed from −20 °C and inactivated by incubation at 56 °C for 30 min, and then diluted in DMEM using a 2-fold serial dilution method, with 50 μL used for each dilution. These dilutions were mixed with 50 μL of rCDV-EGFP virus solution containing approximately 200 TCID_50_. After neutralization at 37 °C for 1 h, approximately 10^5^ Vero cells were added to each well and cultured at 37 °C with 5% CO_2_ for 48 h. GFP expression was observed under a fluorescence microscope. Four replicates were performed for each serum dilution. The number of wells with or without fluorescent spots in each group was recorded, and the serum CDV NAb titer was calculated using the Reed and Muench method.

### 2.11. Mouse Serum Cytokines Detection

The levels of cytokines in the mouse serum samples were measured by using a double antibody sandwich enzyme-linked immunosorbent assay (ELISA) according to instructions from the mouse TNF-α ELISA Kit (Neobioscience, Shenzhen, China), mouse IFN-γ ELISA Kit (Neobioscience, Shenzhen, China), mouse IL-2 ELISA Kit (Invitrogen, Waltham, MA, USA), and mouse IL-4 ELISA Kit (Invitrogen, Waltham, MA, USA), respectively. Mouse sera were subjected to 8-fold dilution and added to 96-well plates pre-coated with specific monoclonal antibodies against mouse TNF-α, IFN-γ, IL-2, or IL-4. After 2 h incubation at room temperature (RT), the plates were then incubated with biotinylated detection antibodies targeting TNF-α, IFN-γ, IL-2 or IL-4 at RT for 1 h. The plates were treated with streptavidin-HRP conjugate for 30 min at RT to amplify the signal, and added substrate solution for colorimetric analysis and 2 M H_2_SO_4_ for reaction stop. Two-wavelength absorbance values of 450 nm/570 nm (OD_450_/OD_570_) were measured by a microplate reader. Mouse cytokine standards in the ELISA kits were simultaneously performed as a parallel control with the standard curve plotting (four-parameter logistic regression fit, R^2^ ≥ 0.95). Concentrations (pg/mL) of different serum cytokines were calculated from the OD_450_/OD_570_ value and standard curve, with final sample concentrations calculated as standard curve readings multiplied by the dilution factor of 8.

### 2.12. Statistical Analysis

The data were analyzed using GraphPad Prism 8.0.1 software (GraphPad Software Inc., San Diego, CA, USA). Statistical differences between groups were assessed using a two-way ANOVA test or a two-tailed unpaired *t* test, with error bars representing the standard error of the mean ± standard error. Statistical comparisons between the assay results were performed, and *p*-values < 0.05 were considered statistically significant (* *p* < 0.05, ** *p* < 0.005, and *** *p* < 0.001).

## 3. Results

### 3.1. The Sequence Source and Analysis of CDV F and H Genes

**Figure 1:**
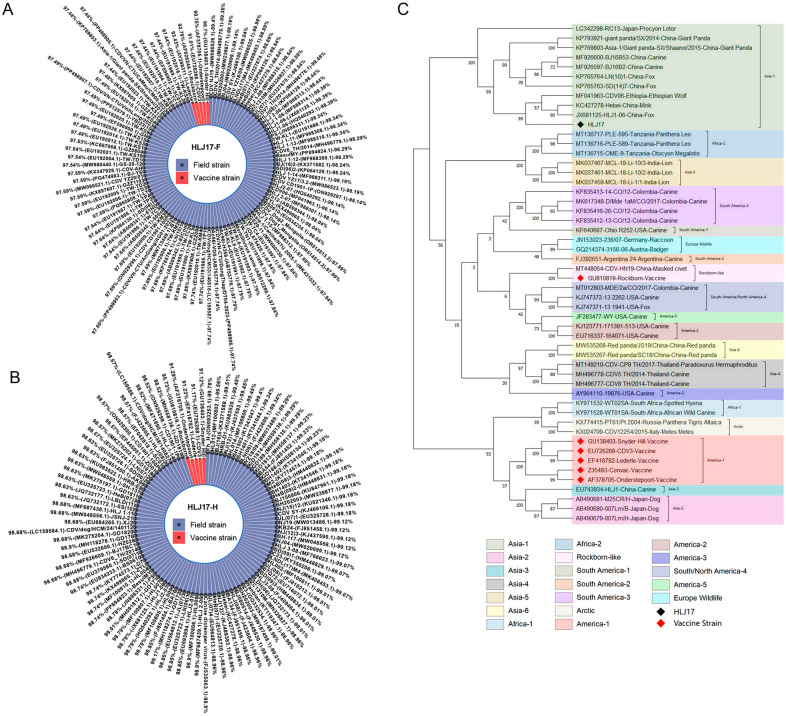
Sequence alignment and phylogenetic tree analysis of the field CDV strain HJL17. (**A**) Homology analysis by BLASTN programs based on the CDV F gene between HJL17 strain and 98 vaccine or field strains in GenBank. (**B**) Homology analysis by BLASTN programs based on the CDV H gene between HJL17 strain and 100 vaccine or field strains in GenBank. (**C**) Phylogenetic tree by MEGA11 software based on the H gene between HLJ17 strain and 51 representative CDV strains of 19 genotypes in GenBank.

A field CDV strain, HLJ17, was isolated from a dog with clinical symptoms of CD. Compared with the five sequences of CDV vaccine strains and 98–100 sequences of CDV field strains in the Genebank, the F gene of HLJ17 strain was 90.7–93.4% genetically identical to that of the vaccine strains, and 97.4–99.4% to that of the field strains (Figure 1A). Similarly, the H gene was 91.1–95.7%, genetically identical to that of the vaccine strains, and 98.5–99.7% to that of the field strains (Figure 1B). The H gene of HJL17 strain was selected to determine the phylogenetic characteristics together with 51 reference sequences, including CDV vaccine strains and field strains derived from different animals. The reference sequences were clustered within 19 known genotypes of CDV, and the HLJ17 strain belonged to the Asia-1 genotype, similar to some filed strains from different species, including the procyon lotor, giant panda, canine, fox, wolf, and mink (Figure 1C). Sequence analysis showed that the HLJ17 F and H gene are different from vaccine strains and similar to the field strains of different species, which have the potential as target genes for vaccine development and may have efficacy in clinical application against the infection of different circulating CDV strains.

### 3.2. Generation of the Recombinant Plasmids Encoding HLJ17H or HLJ17F Gene

**Figure 2:**
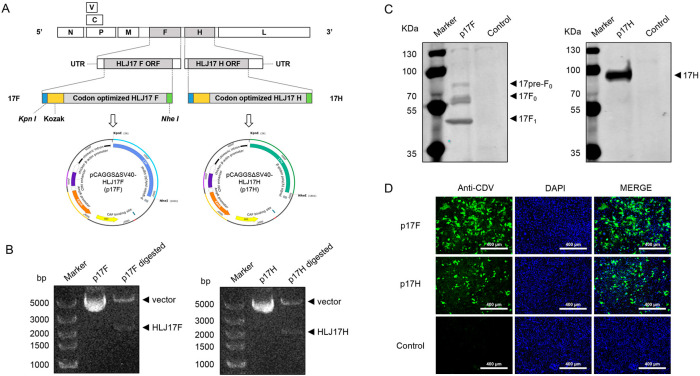
Construction and identification of the recombinant plasmids. (**A**) The schematic diagram of the construction of the recombinant plasmids p17F and p17H. (**B**) Agarose gel electrophoresis of the recombinant plasmids and plasmids digested by the restriction enzymes Kpn I and Nhe I. The plasmids p17F and p17H were digested to expected band of the linearized plasmid pCAGGSΔSV40 (vector) and target genes (HLJ17F and HLJ17H). (**C**) Western blot analysis of the recombinant plasmids p17F and p17H expression. The plasmids p17F and p17H were transfected in HEK-293 cells, and PBS was set as parallel (Control). (**D**) Indirect immunofluorescence analysis of the recombinant plasmids p17F and p17H expression. The plasmids p17F and p17H were transfected in HEK-293 cells, and PBS were set as parallel (Control).

The recombinant plasmids p17F and p17H were generated using the pCAGGSΔSV40 vector platform and genetically modified HLJ17F or HLJ17H (Figure 2A). p17F and p17H were individually double-digested into bands of the expected size (Figure 2B). HEK-293 cells transfected with plasmids p17F and p17H were stained by dog anti-CDV serum (Figure 2C). p17F or p17H expression was further confirmed by western blot analysis with dog anti-CDV serum to CDV F and H proteins (Figure 2D). These results suggested that the recombinant plasmids p17F and p17H were generated, and the target proteins could be efficiently expressed.

### 3.3. Formulation of DNA-LNP Vaccine

**Figure 3:**
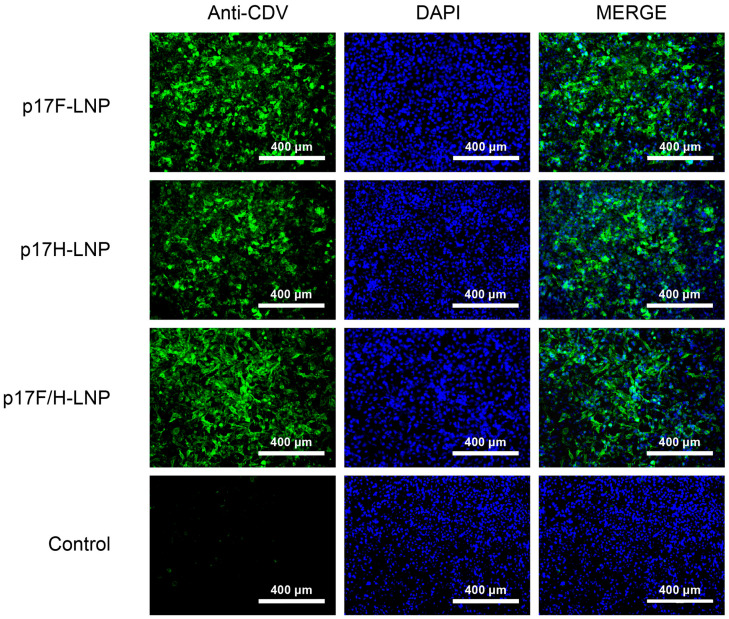
Indirect immunofluorescence analysis of the DNA-LNP formulations. 0.5 μg p17F-LNP, p17H-LNP, or 0.5 μg p17F-LNP and p17H-LNP as bivalent formulations (p17F/H-LNP) were incubated in HEK-293 cells, and PBS was set as parallel (Control).

The p17F-LNP and p17H-LNP were formulated and characterized quality parameters. The concentrations of p17F-LNP and p17H-LNP were above 0.5 mg/mL, and the encapsulation efficiencies were 93.2% and 97.2%, respectively. The nanoparticle diameters of p17F-LNP and p17H-LNP were 105 nm and 104 nm, and the particle diameter indices (PDI) were 0.106 and 0.105, respectively. The cells incubated with p17F-LNP, p17H-LNP, or bivalent formulation were efficiently stained by dog anti-CDV serum (Figure 3), and the in vitro activities were all above 60% based on three independently IFA assays. The results showed that these quality parameters met the standards and could be used as vaccine candidates for further evaluation.

### 3.4. The DNA-LNP Formulations Are Safe and Induce Strong Humoral Immune Responses in Mice

**Figure 4:**
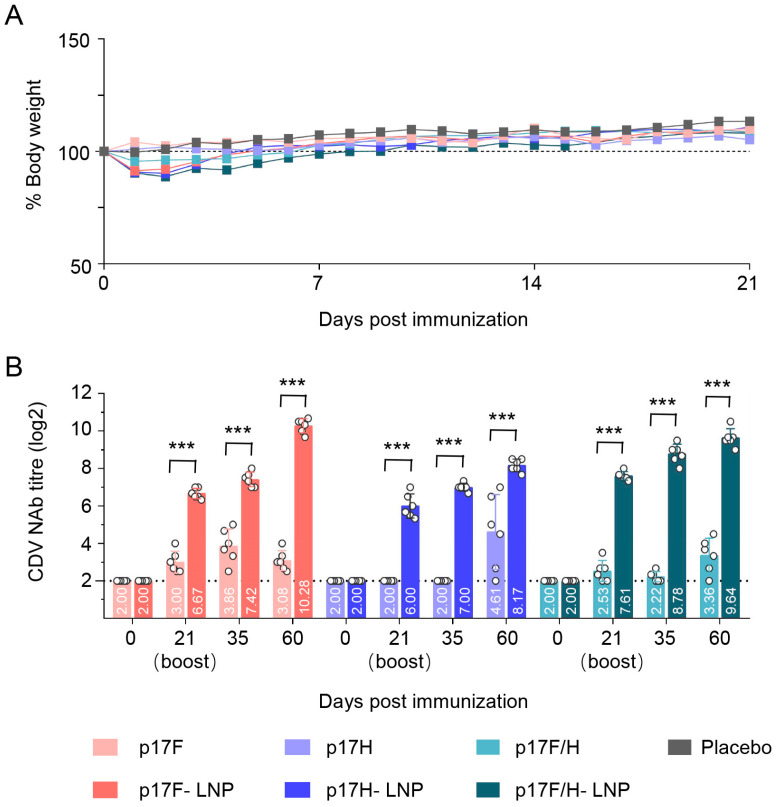
Comparison of safety and immunogenicity evaluation between naked plasmids and DNA-LNP formulations in mice. Mice were intramuscularly immunized with 30 μg of the naked plasmids of p17F, p17H, bivalent formulation (p17F/H), or LNP formulations of p17F-LNP, p17H-LNP, bivalent formulation (p17F/H-LNP) in a 0.1-mL volume twice over 21-day intervals, and PBS was set as parallel (Placebo). Serum CDV neutralizing antibody (NAb) was detected by rCDV-eGFP-based neutralization assays. (**A**) Body weight changes of mice. Body weight changes for groups indicated are shown as ratios of the body weight at day 0, which was set as 100. (**B**) CDV NAb titers of immunized mice at different time points. The horizontal dashed lines indicate the lower limit of detection. Data are presented as mean ± standard error (*n* = 6). Statistical significance was determined by using a two-way ANOVA test in weight changes, and a two-tailed unpaired *t*-test in NAb titers. *** *p* < 0.001.

The DNA-LNP and naked DNA formulations were immunized at the dosage of 30 μg in mice to evaluate the safety and immunogenicity. None of immunized mice exhibited abnormal behavior or clinical symptoms of facial actions, including orbital tightening, nose bulge, change of ear and whisker position, and abnormal states such as red and swollen at the immunization site. All mice immunized with the LNP-encapsulated vaccines experienced no more than 10% weight loss, but had no significant difference in body weight changes compared with the naked DNA-immunized mice and PBS-injected mice during the 21-day observation period (Figure 4A). The plasmids p17F and p17H formulated as DNA-LNP or naked vaccines can induce CDV NAb after prime immunization, and the NAb titers were boosted after the second immunization, with the titers ranged from 8.17 to 10.28 log2 in DNA-LNP formulations or 3.08 to 4.61 log2 in naked formulations at day 60 after prime immunization (Figure 4B). Compared with naked DNA formulation groups, both LNP monovalent and bivalent formulation immunization induced higher NAb levels during the 60-day surveillance period. Moreover, mice immunized with bivalent LNP formulation (p17F/H-LNP) produced superior NAb responses to p17F-LNP and p17H-LNP at days 21 and 35 after primary immunization (Figure 4B). These preliminary results indicated that the DNA-LNP formulations as CDV DNA vaccines were safe, and highly immunogenic in mice.

### 3.5. The DNA-LNP Candidate Vaccines Induce Specific Cytokines Responses and Humoral Immune Responses in Mice

**Figure 5:**
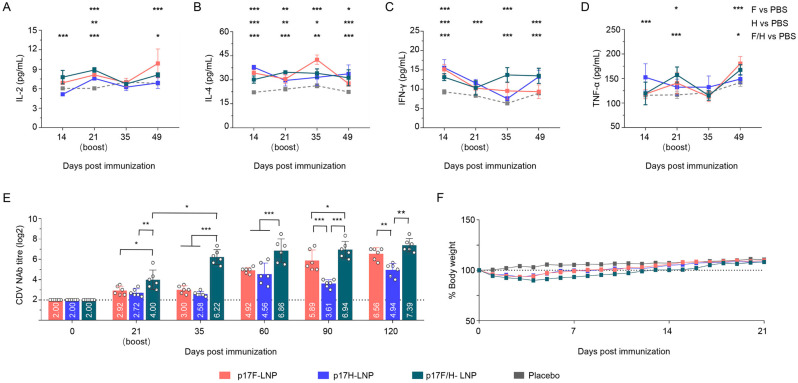
Immunogenicity of DNA-LNP formulations in mice. Mice were intramuscularly immunized with 10 μg of the p17F-LNP, p17H-LNP, bivalent formulation (p17F/H-LNP) in a 0.1-mL volume twice over 21-day intervals, and PBS was set as parallel (Placebo). Serum cytokine production and CDV neutralizing antibody (NAb) at different time points were detected by ELISA and neutralization assays. (**A**) Detection of IL-2 titers. (**B**) Detection of IL-4 titers. (**C**) Detection of IFN-γ titers. (**D**) Detection of TNF-α titers. (**E**) Detection of CDV NAb titers. (**F**) Body weight changes of mice. The horizontal dashed lines indicate the lower limit of detection. Data are presented as mean ± standard error (*n* = 6). Statistical significance was determined by using a two-tailed unpaired *t*-test in NAb titers, and a two-way ANOVA test in weight changes. * *p* < 0.05, ** *p* < 0.01, *** *p* < 0.001.

The DNA-LNP formulations were immunized at the dosage of 10 μg per mouse to determine the safety and immunogenicity of appropriate application dose. Cytokine responses of sera were detected at day 14, 21, 35 and 49 after prime immunization, and significantly higher levels of cytokines compared to the PBS group, including IL-2 (Figure 5A), IL-4 (Figure 5B), IFN-γ (Figure 5C), and TNF-α (Figure 5D). Following the two doses of 10 μg on day 0 and day 21, the DNA-LNP formulations induced NAb responses during the 120-day surveillance period, with titers in p17F-LNP, p17H-LNP, and p17F/H-LNP groups at day 120 after prime immunization were 6.56 log2, 4.94 log2, and 7.39 log2, respectively (Figure 5E). Compared with 17F-LNP or p17H-LNP monovalent formulation, p17F/H-LNP bivalent formulation induced significantly higher levels of NAb responses at different time points after immunization. Moreover, none of immunized mice exhibited abnormal clinical symptoms of facial actions and immunization site, and experienced no significant difference in the body weight changes compared with the PBS-injected mice during the 21-day observation period (Figure 5F). These preliminary results indicated that the p17F/H-LNP formulations with the prime-boost immune program of 10 μg dose and day 21 boost were safe, highly immunogenic in mice.

### 3.6. Immunogenicity of p17F/H-LNP Vaccine in Dogs

**Figure 6:**
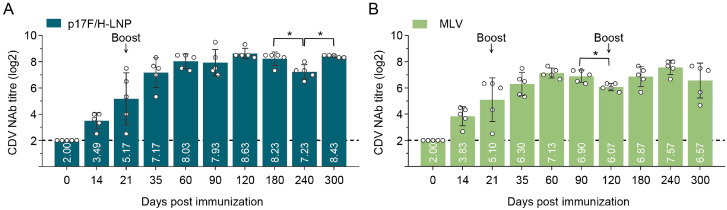
Immunogenicity of p17F/H-LNP formulation in dogs. (**A**) Beagle dogs were intramuscularly immunized with 100 μg of the p17F-LNP and p17H-LNP bivalent formulation (p17F/H-LNP) in a 0.1-mL volume twice over 21-day intervals. (**B**) A commercial CDV vaccine (MLV) was immunized with recommend dose twice over 21-day intervals, and boost at day 120 after prime immunization. Serum CDV neutralizing antibody (NAb) at different time points were detected by neutralization assays. The horizontal dashed lines indicate the lower limit of detection. Data are presented as mean ± standard error (n = 6). Statistical significance was determined by using a two-tailed unpaired *t*-test in NAb titers. * *p* < 0.05.

The p17F/H-LNP formulation was immunized twice over 21-day intervals at the dosage of 100 μg as the applied dose, and a commercial MLV vaccine was immunized by three doses with the recommended dose at days 0, 21 and 120 in dogs. Both p17F/H-LNP (Figure 6A) and MLV (Figure 6B) induced a detectable NAb response in dogs on day 14 after prime immunization. After booster immunization on day 21, the p17F/H-LNP maintained high levels of NAb titer during the immunosurveillance period lasting 300 days, with the titer of 8.63 log2 on day 120 and 8.43 log2 on day 300 after prime immunization (Figure 6A). Similarly, the high level of NAb titer was induced by two doses of MLV, but gradually decreased to 6.07 log2 on day 120 after prime immunization (Figure 6B). The MLV-induced NAb titers were lower than those induced by p17F/H-LNP at the same time points within 120 days after prime immunization, and significantly decreased on day 120 after prime immunization (Figure 6B). To synchronously evaluate the duration of MLV immunogenicity, a third immunization with MLV was performed on day 120 after prime immunization. However, the NAb titer induced by the MLV vaccine began to decrease on day 300 after prime immunization, and remained lower than the p17F/H-LNP vaccine at time points of days 180 and 300 (Figure 6A,B). Furthermore, all dogs vaccinated with p17F/H-LNP and MLV showed no abnormal conditions in clinical behavior, symptoms, and immune sites during the 300-day surveillance period. These results indicated that p17F/H-LNP vaccine has advantages in terms of antibody responses of induction level and duration, and it has the potential to develop canine CD vaccine.

### 3.7. Immunogenicity of p17F/H-LNP Vaccine in Foxes and Raccoon Dogs

**Figure 7:**
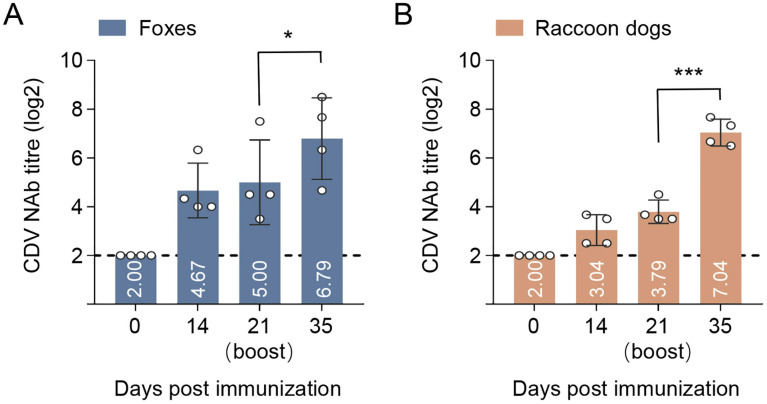
Immunogenicity of p17F/H-LNP formulation in foxes and raccoon dogs. Foxes and raccoon dogs were intramuscularly immunized with 100 μg of the p17F-LNP and p17H-LNP bivalent formulation (p17F/H-LNP) in a 0.1-mL volume twice over 21-day intervals. Serum CDV neutralizing antibody (NAb) at different time points were detected by neutralization assays. (**A**) Detection of CDV NAb titers in foxes. (**B**) Detection of CDV NAb titers in raccoon dogs. The horizontal dashed lines indicate the lower limit of detection. Data are presented as mean ± standard error (n = 4). Statistical significance was determined by using a two-tailed unpaired *t*-test in NAb titers. * *p* < 0.05, *** *p* < 0.001.

To evaluate the immunogenicity in CDV-susceptible wildlife species, the p17F/H-LNP formulation was further immunized in foxes and raccoon dogs at the dosage of 100 μg. The p17F/H-LNP induced NAb response on day 14 after prime immunization, and the titers of NAb were increased on day 21, with the titer of 5.00 log2 in foxes (Figure 7A) and 3.79 log2 in raccoon dogs (Figure 7B). After a secondary immunization on day 21, the p17F/H-LNP could significantly boost the NAb titers both in foxes and raccoon dogs, with the titer of 6.79 log2 in foxes and 7.04 log2 in raccoon dogs on day 35 after prime immunization (Figure 7A,B). In addition, all immunized animals showed no abnormal conditions in clinical behavior, symptoms, and immune sites after the prime-boost immunization. These results indicated that p17F/H-LNP vaccine has expected immunogenicity in foxes and raccoon dogs.

## 4. Discussion

Canine distemper virus (CDV) infection is widely spread worldwide, causing high mortality risk to dogs, foxes, raccoons and minks, which have seen an increase in incidence in recent years [23,24,25,26]. Additionally, fatal infections have been reported in endangered species such as giant pandas, as well as non-human primates such as monkeys and chimpanzees [27,28,29]. Vaccination is an effective approach to CD control. The commercial CD vaccines applied in the field are basically modified live vaccines (MLV), which may have stability problems during production and transportation, as well as safety problems regarding the risk of live virus spillover and transmission, and are less suitable for application in wildlife [30,31]. The continuous mutation of CDV in the field presents new challenges to the clinical efficacy of commercial CDV vaccines. In fact, in clinical applications, some vaccines often suffer from insufficient immune persistence and even pose safety risks in different animals in clinical applications [32,33]. Hence, there is a search for continuous updates and development of safe and effective vaccines. This study aims to develop a promising DNA vaccine based on field epidemic CDV strains and lipid nanoparticles (LNPs) delivery strategies for the industry pain point of developing a novel CD preventive vaccine.

DNA vaccines have attracted much attention in vaccinology as a nucleic acid vaccine technology in the last 30 years, accumulating abundant evidence of promising immunogenicity and protection against virulent challenges for infectious disease prevention, especially in the induction of T-cell responses in non-live vaccines [34]. Compared to MLV, the DNA vaccines demonstrated long-lasting immune effects with a small amount of inoculum, production of cost-effective and simple, and no risk of vaccine virus transmission in production and application [35]. Currently, only a few human or animal DNA vaccines have been successfully approved for application, such as a COVID-19 DNA vaccine for human use in India and an avian influenza DNA vaccine for avian influenza DNA vaccine for poultry use in China [36]. However, DNA vaccine strategies remain with poor immunogenicity or not associated with clinical benefit, in the condition of insufficient immunogenicity of the target antigens for vaccine development. DNA vaccines have been jointly explored in various ways to improve the immunogenicity, including sequence optimization of target genes, optimization of immune programs, selection of administration methods, and application of adjuvants or delivery systems [35]. Here, mammalian codon optimization was chosen to improve the translation efficiency of the HLJ17 F and H gene, and the program of prime-boost immunization was selected to increase the induction level and duration of NAb response. Additionally, the convenient intramuscular injection route was used for animal vaccines considering the efficiency application in animals, without considering other delivery methods such as electroporation (EP) method or microneedles arrays (MNA). More importantly, the LNP delivery system of the approved mRNA vaccine could promote the uptake of nucleic acid by target cells, which acts as an immunoadjuvant, enhancing the delivery efficiency and specific immune responses. Similar reports have been made in the development of vaccines for COVID-19, H1N1 influenza, and African swine fever [37,38,39]. The LNP delivery system in this study follows the process of a commercialized COVID-19 mRNA vaccine [40], which can provide a mature scale-up production process for the future industrialization of this candidate. This study also compared the immunogenicity of a commercialized CDV vaccine, which have a high market share in clinical applications, with trials in dogs, indirectly comparing the previous studies of different vaccines. Therefore, combining these multiple strategies to form a CD DNA-LNP vaccine strategy has excellent development potential.

In field application, the CD vaccines were expected to be effective for over 10 to 12 months. Here, the p17F/H-LNP candidate and a commercial MLV vaccine with high market share were immunized parallel in dogs to evaluate the level and duration of NAb titer induced by two doses immunization for a continuous monitoring period of over 300 days. As expected, the p17F/H-LNP immunized group maintained high levels of NAb during the 300-day monitoring period without a downward trend. However, the MLV immune group showed a significant decrease in NAb titer at 120 days under a two-dose immunization regime. Therefore, we adjusted the immunization strategy to focus on monitoring the duration of immunogenicity of vaccines accordingly. The p17F/H-LNP immunized group still underwent two-dose immunization, while the MLV immunized group boosted a third dose at 120 days to achieve longer-term immune efficacy evaluation without being limited to a two-dose regimen. These preliminary results were as expected, with the two-dose immunization of the p17F/H-LNP inducing the NAb response that lasted for at least 300 days in dogs, and its long immune efficacy may make it advantageous in field application.

The evaluation of challenge protection efficacy is an important indicator of assessing vaccine efficacy. There is a significant correlation between NAb titer induced by CDV vaccine and challenge protection rate. The protection threshold of NAb titer may be affected by the type of vaccine, optimization of the antigen genes, and immunization strategy. In general, prophylactic DNA vaccines for CD could induce relatively low levels of NAb responses, but can efficiently protect animals against virulent challenge [30]. DNA vaccines may initiate immune memory after virulent challenge, rapidly increase NAb levels, and synchronously activate cellular immunity, which can provide efficient challenge protection for dogs [30]. In this study, the p17F/H-LNP candidate induced NAb titer of 6–8 log2 in dogs, foxes and raccoon dogs, and maintained approximately at 8 log2 for at least 300 days in dogs, and specific cytokines responses were shown in the mouse immunization model, indicating that the candidate may have good protective efficacy against virulent challenge. To fulfill the evaluation on p17F/H-LNP as an DNA-LNP vaccine for dogs and other animals, the further research will be extended to animal trials under clinical field conditions. These studies will include evaluation of cross-neutralization efficiency for other field CDV genotypes, longer duration monitoring of NAb responses in animals, and animal challenge protection tests.

The other important issue with vaccines is safety, and this needs more comprehensive evaluation of the DNA-LNP vaccines based on the LNP delivery system of mRNA vaccines. Although human mRNA vaccines have been widely used in recent years, safety remains slightly controversial due to the application of LNP delivery systems [41]. The LNP delivery system of p17F/H-LNP in this study is homologous to the first COVID-19 mRNA vaccine in China, using a four-component prescription design, among which the cationic liposome is SM-102, has a low risk of inflammatory storm caused by vaccination, which is the promising choice recognized by the industry [40,42]. Mice immunized with 30 μg p17F/H-LNP showed abnormal clinical conditions, such as toxicological indicators, including orbital tightness, nose bulge, cheek bulge, ear posture and whiskers. Although the mice lost weight slightly in the first five days after prime immunization, they recovered quickly and grew steadily without any growth retardation or abnormal fluctuations. Moreover, dogs, foxes and raccoon dogs showed no abnormal conditions, including mental state, diet and water drinking, respiratory frequency, and no inflammatory reactions such as redness, swelling at the injection site after prime-boost immunization. These studies indicated that the p17F/H-LNP candidates showed good biological safety in mice, dogs, foxes, and raccoon dogs, and did not cause acute or systemic toxicity. Admittedly, whether the p17F/H-LNP vaccines are safe for different animals remains a subject for further investigation before the field studies. We plan to conduct intermediate trials of this DNA-vaccine candidate according the requirements of Genetically Modified Organisms (GMO) safety evaluation, which will include the evaluation of the metabolic dynamics and retention time of the LNP formulation at injection sites in animals and throughout the body, the activation status of toxicity and inflammatory pathways, and the effects of the LNP formulation on injection sites, liver, and kidney tissues through pathological sections and immunohistochemistry.

## 5. Conclusions

This study developed a p17F/H-LNP candidate vaccine based on the F and H proteins of CDV HLJ17 strain, demonstrating strong immunogenicity in mice, dogs, foxes, and raccoon dogs. The p17F/H-LNP vaccine induced high NAb levels, with immunity lasting over 300 days in dogs after two doses, outperforming traditional vaccines. LNP encapsulation significantly enhanced immune responses, and the combined F and H antigens exhibited synergistic effects. These preliminary findings highlight the DNA-LNP vaccine as a promising strategy for CDV prevention in both domestic and wild animals.

## Data Availability

Data are contained within the article.
